# Supplementary material for: A Likelihood Approach for Real-Time Calibration of Stochastic Compartmental Epidemic Models
Source: PLoS Comput Biol. 2017 Jan 17;13(1):e1005257. doi: 10.1371/journal.pcbi.1005257 (PMC5240920; doi:10.1371/journal.pcbi.1005257)
Supplement: S1 File — (TAR.GZ) [file pcbi.1005257.s014.tar.gz › HSPH_Online-SI-Revision/PF08/n10-severe/n10-severe_PF2_table8.pdf]

1	newly 3 weeks spec	53.	135.89	{113., 158.}	156.396	0.404562	newly 3 weeks cumul	226.	494.11	{427., 572.}	118.633	0.336678	newly 5 weeks spec	40.	97.07	{78., 119.}	142.675	0.379488	newly 5 weeks cumul	307.	707.01	{617., 822.}	130.296	0.359218	newly 43 weeks cumul	452.	1154.55	{943., 1382.}	155.431	0.402725
2	newly 3 weeks spec	48.	164.61	{143., 187.}	242.938	0.532494	newly 3 weeks cumul	239.	594.62	{539., 660.}	148.795	0.394271	newly 5 weeks spec	25.	109.48	{91., 128.}	337.92	0.637488	newly 5 weeks cumul	302.	837.98	{755., 927.}	177.477	0.441665	newly 45 weeks cumul	375.	1301.61	{1134., 1492.}	247.096	0.538026
3	newly 3 weeks spec	7.	137.03	{115., 162.}	1857.57	1.28819	newly 3 weeks cumul	64.	508.36	{463., 579.}	694.313	0.898446	newly 5 weeks spec	1.	91.57	{74., 118.}	9057.	1.95405	newly 5 weeks cumul	67.	713.37	{648., 820.}	964.731	1.02532	newly 47 weeks cumul	68.	1070.85	{937., 1253.}	1474.78	1.19407
4	newly 3 weeks spec	37.	147.51	{124., 168.}	298.676	0.597733	newly 3 weeks cumul	140.	520.28	{466., 555.}	271.629	0.569172	newly 5 weeks spec	17.	105.53	{88., 122.}	520.765	0.789437	newly 5 weeks cumul	186.	752.89	{676., 802.}	304.78	0.606254	newly 42 weeks cumul	207.	1283.67	{1145., 1399.}	520.13	0.790911
5	newly 3 weeks spec	63.	149.73	{121., 172.}	137.667	0.371819	newly 3 weeks cumul	261.	533.13	{465., 596.}	104.264	0.308167	newly 5 weeks spec	22.	107.46	{80., 134.}	388.455	0.682707	newly 5 weeks cumul	329.	769.87	{667., 861.}	134.003	0.367062	newly 42 weeks cumul	383.	1309.69	{1033., 1521.}	241.956	0.530087
6	newly 3 weeks spec	37.	156.53	{127., 189.}	323.054	0.621828	newly 3 weeks cumul	120.	549.62	{475., 623.}	358.017	0.658622	newly 5 weeks spec	13.	113.95	{92., 139.}	776.538	0.93695	newly 5 weeks cumul	158.	798.79	{675., 909.}	405.563	0.701299	newly 43 weeks cumul	173.	1395.92	{1114., 1647.}	706.89	0.90236
7	newly 3 weeks spec	17.	99.27	{75., 132.}	483.941	0.755679	newly 3 weeks cumul	136.	352.7	{285., 437.}	159.338	0.406998	newly 5 weeks spec	12.	72.11	{50., 102.}	500.917	0.763228	newly 5 weeks cumul	163.	510.77	{414., 635.}	213.356	0.488506	newly 42 weeks cumul	196.	891.77	{668., 1188.}	354.985	0.646191
8	newly 3 weeks spec	10.	142.19	{114., 167.}	1321.9	1.14817	newly 3 weeks cumul	74.	513.33	{426., 578.}	593.689	0.8385	newly 5 weeks spec	3.	99.04	{76., 120.}	3201.33	1.5122	newly 5 weeks cumul	80.	731.69	{614., 813.}	814.613	0.958377	newly 46 weeks cumul	83.	1158.76	{959., 1330.}	1296.1	1.14121
9	newly 3 weeks spec	28.	160.7	{137., 184.}	473.929	0.755205	newly 3 weeks cumul	137.	607.72	{553., 680.}	343.591	0.645542	newly 5 weeks spec	9.	97.32	{75., 118.}	981.333	1.02716	newly 5 weeks cumul	177.	829.36	{733., 936.}	368.565	0.669065	newly 50 weeks cumul	178.	1149.52	{985., 1338.}	545.798	0.807341
10	newly 3 weeks spec	9.	130.98	{109., 151.}	1355.33	1.15974	newly 3 weeks cumul	56.	503.54	{463., 546.}	799.179	0.952959	newly 5 weeks spec	0.1	81.99	{65., 97.}	81 890.	2.90865	newly 5 weeks cumul	64.	689.3	{631., 749.}	977.031	1.03127	newly 50 weeks cumul	67.	955.64	{839., 1058.}	1326.33	1.15239
11	newly 3 weeks spec	0.1	150.49	{133., 164.}	150 390.	3.17586	newly 3 weeks cumul	7.	575.1	{548., 601.}	8115.71	1.91441	newly 5 weeks spec	0.1	91.39	{80., 105.}	91 290.	2.95845	newly 5 weeks cumul	7.	784.62	{756., 819.}	11 108.9	2.04932	newly 49 weeks cumul	7.	1097.59	{1044., 1158.}	15 579.9	2.19499
12	newly 3 weeks spec	18.	180.13	{157., 204.}	900.722	0.998436	newly 3 weeks cumul	97.	656.07	{597., 703.}	576.361	0.829392	newly 5 weeks spec	11.	119.92	{99., 138.}	990.182	1.03403	newly 5 weeks cumul	121.	919.57	{842., 994.}	659.975	0.879948	newly 49 weeks cumul	154.	1373.29	{1211., 1514.}	791.747	0.948527
13	newly 3 weeks spec	27.	148.7	{127., 164.}	450.741	0.738546	newly 3 weeks cumul	156.	557.61	{507., 618.}	257.442	0.551972	newly 5 weeks spec	15.	98.49	{82., 115.}	556.6	0.813558	newly 5 weeks cumul	190.	779.33	{711., 863.}	310.174	0.611827	newly 46 weeks cumul	207.	1169.46	{1056., 1303.}	464.957	0.75057
14	newly 3 weeks spec	5.	144.05	{126., 161.}	2781.	1.45726	newly 3 weeks cumul	40.	523.16	{485., 560.}	1207.9	1.11563	newly 5 weeks spec	0.1	96.34	{77., 115.}	96 240.	2.97897	newly 5 weeks cumul	41.	737.56	{669., 794.}	1698.93	1.25394	newly 48 weeks cumul	41.	1142.9	{1021., 1254.}	2687.56	1.44348
15	newly 3 weeks spec	2.	102.87	{84., 121.}	5043.5	1.70707	newly 3 weeks cumul	22.	421.23	{385., 455.}	1814.68	1.28122	newly 5 weeks spec	2.	57.55	{43., 72.}	2777.5	1.45129	newly 5 weeks cumul	26.	556.14	{497., 606.}	2039.	1.32913	newly 52 weeks cumul	28.	712.29	{630., 789.}	2443.89	1.40391
16	newly 3 weeks spec	8.	82.41	{65., 102.}	930.125	1.00585	newly 3 weeks cumul	58.	324.2	{276., 366.}	458.966	0.744063	newly 5 weeks spec	4.	48.37	{32., 66.}	1109.25	1.06565	newly 5 weeks cumul	73.	435.97	{359., 510.}	497.219	0.771815	newly 48 weeks cumul	90.	589.25	{457., 729.}	554.722	0.809027
17	newly 3 weeks spec	89.	180.6	{147., 211.}	103.124	0.302561	newly 3 weeks cumul	412.	648.99	{566., 742.}	58.3471	0.197385	newly 5 weeks spec	62.	119.6	{100., 141.}	93.0645	0.279691	newly 5 weeks cumul	564.	915.71	{795., 1049.}	63.1294	0.210142	newly 48 weeks cumul	769.	1409.48	{1202., 1691.}	84.0546	0.261022
18	newly 3 weeks spec	67.	153.	{120., 187.}	128.358	0.352729	newly 3 weeks cumul	293.	561.63	{459., 649.}	91.6826	0.279117	newly 5 weeks spec	37.	101.7	{78., 123.}	174.865	0.431996	newly 5 weeks cumul	379.	786.85	{635., 908.}	107.612	0.313617	newly 46 weeks cumul	562.	1201.86	{943., 1425.}	113.854	0.325283
19	newly 3 weeks spec	35.	143.5	{124., 164.}	310.	0.610643	newly 3 weeks cumul	159.	511.83	{470., 556.}	221.906	0.506677	newly 5 weeks spec	32.	102.08	{87., 115.}	219.	0.500524	newly 5 weeks cumul	221.	734.35	{673., 797.}	232.285	0.520458	newly 42 weeks cumul	292.	1219.06	{1094., 1342.}	317.486	0.618986
20	newly 3 weeks spec	92.	169.82	{148., 192.}	84.587	0.264174	newly 3 weeks cumul	401.	572.07	{525., 610.}	42.6608	0.153577	newly 5 weeks spec	59.	129.36	{110., 149.}	119.254	0.337903	newly 5 weeks cumul	544.	850.72	{779., 905.}	56.3824	0.193416	newly 42 weeks cumul	745.	1627.12	{1441., 1792.}	118.405	0.337684
21	newly 3 weeks spec	36.	163.91	{139., 187.}	355.306	0.655337	newly 3 weeks cumul	108.	597.81	{548., 645.}	453.528	0.742157	newly 5 weeks spec	14.	103.58	{85., 122.}	639.857	0.864889	newly 5 weeks cumul	144.	829.51	{752., 904.}	476.049	0.759368	newly 50 weeks cumul	181.	1211.77	{1054., 1328.}	569.486	0.824257
22	newly 3 weeks spec	13.	134.34	{108., 163.}	933.385	1.00881	newly 3 weeks cumul	94.	489.9	{436., 558.}	421.17	0.714672	newly 5 weeks spec	3.	91.54	{69., 114.}	2951.33	1.47483	newly 5 weeks cumul	123.	693.41	{601., 796.}	463.748	0.748189	newly 48 weeks cumul	123.	1064.72	{846., 1252.}	765.626	0.931882
23	newly 3 weeks spec	73.	134.49	{97., 177.}	84.2329	0.254443	newly 3 weeks cumul	250.	490.61	{382., 641.}	96.244	0.285138	newly 5 weeks spec	30.	92.88	{66., 125.}	209.6	0.475989	newly 5 weeks cumul	319.	696.11	{527., 906.}	118.216	0.330371	newly 47 weeks cumul	375.	1097.89	{805., 1473.}	192.771	0.454243
24	newly 3 weeks spec	15.	148.87	{129., 164.}	892.467	0.995089	newly 3 weeks cumul	84.	573.14	{541., 604.}	582.31	0.833607	newly 5 weeks spec	11.	91.66	{78., 105.}	733.273	0.917549	newly 5 weeks cumul	110.	782.41	{746., 815.}	611.282	0.851717	newly 48 weeks cumul	115.	1102.62	{1038., 1164.}	858.8	0.981154
25	newly 3 weeks spec	0.1	143.64	{129., 160.}	143 540.	3.15547	newly 3 weeks cumul	2.	573.66	{545., 625.}	28 583.	2.45713	newly 5 weeks spec	0.1	84.67	{71., 97.}	84 570.	2.92478	newly 5 weeks cumul	2.	769.17	{739., 845.}	38 358.5	2.58447	newly 51 weeks cumul	2.	1037.01	{992., 1173.}	51 750.5	2.71407
26	newly 3 weeks spec	24.	182.55	{159., 208.}	660.625	0.878631	newly 3 weeks cumul	102.	664.4	{609., 727.}	551.373	0.812784	newly 5 weeks spec	5.	118.79	{97., 138.}	2275.8	1.37135	newly 5 weeks cumul	124.	930.67	{842., 1037.}	650.54	0.874144	newly 50 weeks cumul	128.	1393.82	{1230., 1621.}	988.922	1.03476
27	newly 3 weeks spec	35.	185.42	{159., 212.}	429.771	0.720967	newly 3 weeks cumul	192.	697.05	{616., 778.}	263.047	0.558559	newly 5 weeks spec	19.	115.53	{93., 137.}	508.053	0.778899	newly 5 weeks cumul	236.	957.03	{837., 1067.}	305.521	0.606405	newly 51 weeks cumul	284.	1339.11	{1143., 1548.}	371.518	0.670984
28	newly 3 weeks spec	7.	232.05	{202., 256.}	3215.	1.51872	newly 3 weeks cumul	31.	835.23	{779., 885.}	2594.29	1.42987	newly 5 weeks spec	4.	154.92	{128., 180.}	3773.	1.58443	newly 5 weeks cumul	37.	1179.06	{1083., 1262.}	3086.65	1.50261	newly 50 weeks cumul	43.	1779.74	{1620., 1925.}	4038.93	1.61576
29	newly 3 weeks spec	0.1	134.01	{113., 154.}	133 910.	3.12411	newly 3 weeks cumul	12.	521.28	{487., 565.}	4244.	1.63718	newly 5 weeks spec	0.1	78.79	{63., 93.}	78 690.	2.89162	newly 5 weeks cumul	12.	702.85	{643., 765.}	5757.08	1.76684	newly 52 weeks cumul	12.	952.93	{849., 1073.}	7841.08	1.89824
30	newly 3 weeks spec	0.1	113.99	{99., 129.}	113 890.	3.05461	newly 3 weeks cumul	4.	463.08	{444., 484.}	11 477.	2.06324	newly 5 weeks spec	0.1	64.37	{53., 74.}	64 270.	2.8046	newly 5 weeks cumul	4.	613.03	{586., 630.}	15 225.8	2.18511	newly 52 weeks cumul	4.	803.95	{770., 831.}	19 998.8	2.30281
31	newly 3 weeks spec	16.	134.1	{113., 158.}	738.125	0.91892	newly 3 weeks cumul	78.	507.72	{448., 557.}	550.923	0.811116	newly 5 weeks spec	25.	88.14	{70., 108.}	252.56	0.538053	newly 5 weeks cumul	122.	706.94	{623., 779.}	479.459	0.760025	newly 49 weeks cumul	141.	1031.26	{857., 1196.}	631.39	0.859249
32	newly 3 weeks spec	66.	129.69	{88., 159.}	96.6818	0.28329	newly 3 weeks cumul	192.	446.99	{326., 535.}	132.807	0.358864	newly 5 weeks spec	25.	97.67	{61., 128.}	290.68	0.577028	newly 5 weeks cumul	260.	657.5	{460., 789.}	152.885	0.393571	newly 42 weeks cumul	338.	1209.62	{740., 1568.}	257.876	0.537988
33	newly 3 weeks spec	53.	155.61	{125., 186.}	193.604	0.463449	newly 3 weeks cumul	200.	539.16	{466., 608.}	169.58	0.428553	newly 5 weeks spec	33.	114.11	{88., 145.}	245.788	0.531339	newly 5 weeks cumul	277.	788.46	{676., 892.}	184.643	0.451642	newly 42 weeks cumul	389.	1398.62	{1099., 1682.}	259.542	0.550651
34	newly 3 weeks spec	62.	137.97	{108., 168.}	122.532	0.341215	newly 3 weeks cumul	247.	476.58	{384., 555.}	92.9474	0.281428	newly 5 weeks spec	32.	96.15	{73., 121.}	200.469	0.469896	newly 5 weeks cumul	321.	687.45	{552., 797.}	114.159	0.326232	newly 42 weeks cumul	401.	1171.42	{906., 1401.}	192.125	0.458775
35	newly 3 weeks spec	19.	202.18	{175., 230.}	964.105	1.02466	newly 3 weeks cumul	121.	717.19	{656., 775.}	492.719	0.771978	newly 5 weeks spec	16.	138.52	{115., 165.}	765.75	0.933597	newly 5 weeks cumul	158.	1021.75	{925., 1113.}	546.677	0.809698	newly 49 weeks cumul	183.	1590.36	{1425., 1774.}	769.049	0.937478
36	newly 3 weeks spec	68.	157.51	{112., 195.}	131.632	0.356277	newly 3 weeks cumul	286.	574.53	{431., 688.}	100.885	0.296537	newly 5 weeks spec	63.	110.95	{73., 146.}	76.3968	0.234156	newly 5 weeks cumul	404.	820.85	{602., 1002.}	103.181	0.300785	newly 45 weeks cumul	496.	1322.3	{924., 1684.}	166.593	0.415627
37	newly 3 weeks spec	40.	134.84	{110., 161.}	237.1	0.523159	newly 3 weeks cumul	178.	495.15	{440., 562.}	178.174	0.442325	newly 5 weeks spec	13.	92.04	{74., 112.}	608.	0.844243	newly 5 weeks cumul	219.	701.19	{618., 796.}	220.178	0.503358	newly 48 weeks cumul	249.	1084.05	{933., 1265.}	335.361	0.635612
38	newly 3 weeks spec	60.	165.55	{125., 208.}	175.917	0.433246	newly 3 weeks cumul	237.	604.63	{468., 727.}	155.118	0.401271	newly 5 weeks spec	33.	106.58	{79., 134.}	222.97	0.500833	newly 5 weeks cumul	316.	845.16	{660., 1012.}	167.456	0.421766	newly 48 weeks cumul	375.	1280.38	{958., 1570.}	241.435	0.526185
39	newly 3 weeks spec	66.	160.42	{116., 204.}	143.061	0.376162	newly 3 weeks cumul	292.	561.46	{443., 681.}	92.2808	0.277512	newly 5 weeks spec	58.	114.87	{75., 145.}	98.1897	0.285492	newly 5 weeks cumul	396.	814.28	{622., 996.}	105.626	0.305835	newly 44 weeks cumul	641.	1394.08	{975., 1737.}	117.485	0.328278
40	newly 3 weeks spec	57.	162.2	{124., 190.}	184.561	0.448338	newly 3 weeks cumul	264.	581.15	{469., 657.}	120.133	0.338228	newly 5 weeks spec	47.	112.19	{82., 136.}	138.702	0.369983	newly 5 weeks cumul	347.	827.1	{656., 956.}	138.357	0.372429	newly 44 weeks cumul	489.	1355.64	{990., 1605.}	177.227	0.435117
41	newly 3 weeks spec	16.	131.82	{117., 148.}	723.875	0.91385	newly 3 weeks cumul	51.	532.01	{502., 563.}	943.157	1.01782	newly 5 weeks spec	0.1	79.57	{67., 92.}	79 470.	2.89729	newly 5 weeks cumul	55.	716.25	{680., 750.}	1202.27	1.11412	newly 52 weeks cumul	59.	971.01	{917., 1018.}	1545.78	1.21567
42	newly 3 weeks spec	25.	154.74	{137., 174.}	518.96	0.789895	newly 3 weeks cumul	108.	557.01	{533., 577.}	415.75	0.712033	newly 5 weeks spec	2.	109.55	{95., 126.}	5377.5	1.73612	newly 5 weeks cumul	125.	796.84	{759., 831.}	537.472	0.804109	newly 43 weeks cumul	125.	1285.28	{1219., 1368.}	928.224	1.01152
43	newly 3 weeks spec	9.	155.65	{139., 172.}	1629.44	1.23609	newly 3 weeks cumul	69.	589.31	{556., 619.}	754.072	0.931035	newly 5 weeks spec	6.	95.35	{82., 109.}	1489.17	1.19841	newly 5 weeks cumul	83.	806.57	{758., 836.}	871.771	0.98716	newly 51 weeks cumul	83.	1141.55	{1058., 1218.}	1275.36	1.13773
44	newly 3 weeks spec	52.	150.55	{115., 184.}	189.519	0.45466	newly 3 weeks cumul	260.	538.49	{443., 632.}	107.112	0.312003	newly 5 weeks spec	27.	106.14	{78., 133.}	293.111	0.585361	newly 5 weeks cumul	320.	769.89	{619., 919.}	140.591	0.376596	newly 45 weeks cumul	421.	1264.62	{982., 1586.}	200.385	0.470277
45	newly 3 weeks spec	23.	137.16	{109., 163.}	496.348	0.770213	newly 3 weeks cumul	107.	516.73	{423., 591.}	382.925	0.680856	newly 5 weeks spec	11.	88.54	{69., 106.}	704.909	0.899618	newly 5 weeks cumul	130.	715.93	{589., 816.}	450.715	0.737777	newly 48 weeks cumul	143.	1049.36	{842., 1199.}	633.818	0.861615
46	newly 3 weeks spec	21.	137.93	{106., 187.}	556.81	0.808589	newly 3 weeks cumul	150.	490.06	{414., 650.}	226.707	0.508796	newly 5 weeks spec	19.	95.02	{69., 141.}	400.105	0.684946	newly 5 weeks cumul	189.	701.35	{579., 930.}	271.085	0.56318	newly 47 weeks cumul	195.	1131.25	{860., 1548.}	480.128	0.752783
47	newly 3 weeks spec	36.	145.95	{115., 175.}	305.417	0.60079	newly 3 weeks cumul	193.	526.83	{421., 591.}	172.969	0.432475	newly 5 weeks spec	17.	98.97	{74., 121.}	482.176	0.754855	newly 5 weeks cumul	233.	749.29	{594., 852.}	221.584	0.50292	newly 48 weeks cumul	246.	1167.51	{898., 1399.}	374.598	0.669034
48	newly 3 weeks spec	58.	167.01	{140., 195.}	187.948	0.455635	newly 3 weeks cumul	260.	606.39	{545., 688.}	133.227	0.365659	newly 5 weeks spec	33.	111.67	{88., 132.}	238.394	0.524438	newly 5 weeks cumul	358.	852.71	{749., 959.}	138.187	0.374655	newly 45 weeks cumul	432.	1331.57	{1169., 1535.}	208.234	0.485789
49	newly 3 weeks spec	33.	124.62	{102., 147.}	277.636	0.570987	newly 3 weeks cumul	141.	461.12	{405., 526.}	227.035	0.510629	newly 5 weeks spec	29.	81.37	{59., 106.}	180.586	0.436343	newly 5 weeks cumul	202.	647.33	{562., 762.}	220.46	0.5011	newly 47 weeks cumul	230.	986.91	{813., 1202.}	329.091	0.625482
50	newly 3 weeks spec	0.1	61.27	{46., 74.}	61 170.	2.78012	newly 3 weeks cumul	33.	258.16	{218., 298.}	682.303	0.889982	newly 5 weeks spec	0.1	31.33	{20., 41.}	31 230.	2.47891	newly 5 weeks cumul	33.	332.76	{273., 384.}	908.364	0.99962	newly 52 weeks cumul	33.	409.99	{328., 488.}	1142.39	1.08869
